# A concise flow synthesis of indole-3-carboxylic ester and its derivatisation to an auxin mimic

**DOI:** 10.3762/bjoc.13.251

**Published:** 2017-11-29

**Authors:** Marcus Baumann, Ian R Baxendale, Fabien Deplante

**Affiliations:** 1 Department of Chemistry, University of Durham, South Road, Durham, Durham, DH1 3LE, UK

**Keywords:** flow chemistry, heterocycle, hydrogenation, indole, multistep

## Abstract

An assembled suite of flow-based transformations have been used to rapidly scale-up the production of a novel auxin mimic-based herbicide which was required for preliminary field trials. The overall synthetic approach and optimisation studies are described along with a full description of the final reactor configurations employed for the synthesis as well as the downstream processing of the reaction streams.

## Introduction

**Figure 1:**
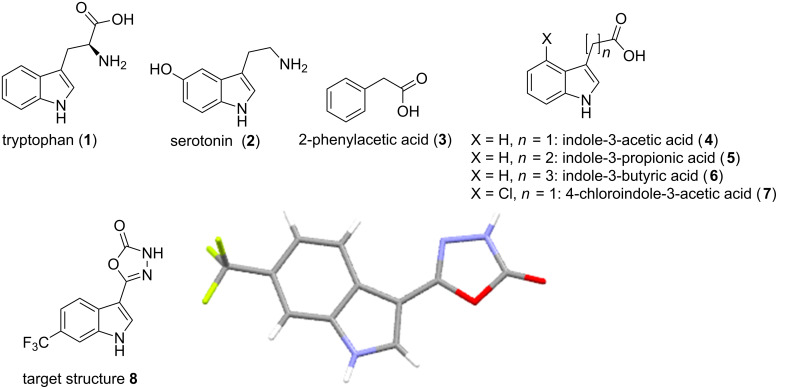
Natural indole containing molecules **1**–**7** of biological importance and synthetic auxin analogue **8** required at scale and X-ray structure.

Indoles are amongst the most important bioactive heterocyclic structures being commonly encountered in the amino acid tryptophan (**1**), the related neurotransmitter serotonin (**2**) as well as numerous complex alkaloid natural products and pharmaceuticals [1–4]. Indoles also play a significant role as phytohormones that promote and regulate the growth and development of plants. Indeed, four of the five endogenously synthesised auxins produced by plants contain the indole motif (Figure 1, structures **3**–**7**). As a consequence of their regulatory activity these structures have become prime targets for investigations into both enhancing plant growth as well as targeted plant growth inhibition generating new agrochemical herbicides [5]. A recent collaboration investigating the uptake and resulting distribution of synthetic indole-3-acetic acid analogues in broad leaf plant species (dicotyledons) required the preparation of structure **8** (Figure 1) at scale for extended field trials. Furthermore as weather patterns and environmental concerns impact significantly on the timing and ultimately the quantities of material required in such studies it was also deemed highly desirable to be able to produce material on demand using a flexible scale flow chemistry approach.

Since the report of the first synthetic access to indoles by Fischer in 1835 more than a dozen further unique indole syntheses have been reported showcasing the importance of developing new entries into these valuable structures [6]. However, common to the majority of these indole syntheses is the use of hazardous entities such as hydrazines (Fischer), diazonium species (Japp–Klingemann) or azides (Hemetsberger–Knittel) or the necessity to construct specifically functionalised precursors in a multistep sequence prior to indole ring formation [7]. In order to address these potential shortcomings we set out to develop a benign process relying on inexpensive substrates and non-toxic reagents that would rapidly deliver the desired indole in a readily scalable and continuous fashion.

## Results and Discussion

**Scheme 1:**
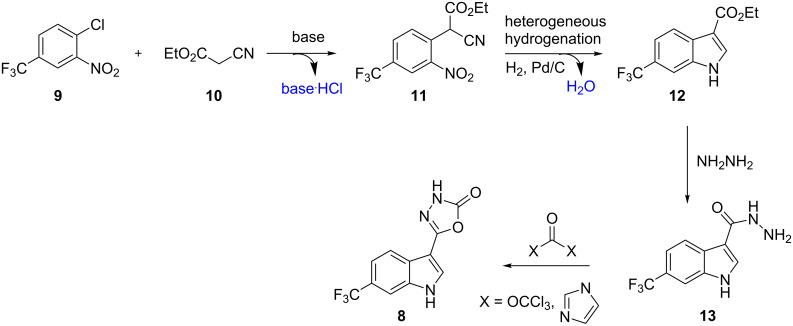
Synthetic strategy towards desired indole product **8**.

In order to generate the core indole unit through a robust synthetic sequence we decided to investigate the treatment of a 2-chloronitrobenzene **9** with ethyl cyanoacetate (**10**) as the nucleophile in a base-mediated S_N_Ar reaction (Scheme 1). The resulting adduct **11** would then be subjected to heterogeneous hydrogenation conditions to produce the indole product **12** through a reductive cyclisation sequence. From the corresponding ester functionalised indole **12** we anticipated that condensation with hydrazine would furnish the corresponding acyl hydrazine **13** which could be cyclised to the desired product **8** through the action of a reactive carbonyl donor such as CDI (1,1′-carbonyldiimidazole) or triphosgene (bis(trichloromethyl)carbonate). This synthetic strategy presented various advantages as it relies on readily available substrates and reagents, creates small amounts of non-toxic byproducts (base**·**HCl, H_2_O) and uses industrially favourable hydrogenation protocols in the key cyclisation step.

To commence the study we first conducted a comprehensive screening program to determine flow compatible conditions for the formation of compound **11** optimising for solvent, base, temperature and reagent stoichiometry – selected results are presented in Table 1.

**Table 1:** Optimisation experiments for S_N_Ar with ethyl cyanoacetate (**10**).^a^

Entry	**9** [M]	**10** (equiv)	Solvent^b^	Base^c^ (equiv)	Temp. (°C)	% Conv.

1	0.25	2	MeCN	Et_3_N (5)	50	8
2	0.25	2	DCM	Et_3_N (5)	40	3
3	0.25	2	DMF	Et_3_N (5)	70	6
4	0.5	2	DMF	TMG (3)	40	98
5	0.75	2	DMF	TMG (3)	40	100^d^
6	0.25	1	DMF	TMG (3)	40	88
7	0.25	2	DCM	TMG (3)	40	100
8	0.25	2	EtOH	NaOEt (3)	40	–^e^
9	0.25	2	*t*-BuOH	KO*t*-Bu	40	44^d^
10	0.25	2	DMF	K_2_CO_3_ (5)	40	100^d^
11	0.25	2	THF	TMG (3)	50	55^d^
12	0.25	2	EtOAc	TMG (3)	50	90^d^
13	0.25	2	EtOAc/MeCN 5:1	TMG (2.5)	50	>98
14	0.25	2	EtOH	TMG (3)	50	64
15	0.25	2	MeCN	TMG (3)	50	100
16	0.25	1	MeCN	TMG (3)	50	90
17	0.25	1.5	MeCN	TMG (3)	50	100
18	0.25	1.2	MeCN	TMG (3)	50	100
19	0.25	1.1	MeCN	TMG (3)	50	100
20	0.25	1.1	MeCN	TMG (2)	50	87
21	0.25	1.1	MeCN	TMG (2.2)	50	94
22	0.25	1.1	MeCN	TMG (2.5)	50	100
23	0.25	1.1	MeCN	TMG (2.5)	40	96
24	0.25	1.1	DCM	TMG (2.5)	50	100
25	0.25	1.1	DCM	TMG (2.5)	40	89
26	0.5	1.1	DMF	TMG (2.5)	50	100
27	0.25	2	EtOAc/MeCN 5:1	TMG (2.5)	50	100

^a^All reactions were run in Biotage microwave vials being irradiated at the specified temperature for 1 hour. The organic phase was analysed after work-up by quenching with 1 M HCl (and extraction with EtOAc if required), drying over anhydrous Na_2_SO_4_ and solvent evaporation. Conversion to product was based upon calibrated LC. ^b^Solvents were tested for full solubility of reagents and for the deprotonated ethyl cyanoacetate (**10**) prior to full testing. ^c^It was determined that DBU (1,8-diazabicyclo[5.4.0]undec-7-ene) and TMG (1,1,3,3-tetramethylguanidine) could be used interchangeably without any effect on the yield or product purity, for clarity only the results with TMG are shown. ^d^Solid formation occurred during the reaction. ^e^Complex mixture generated including direct addition of the ethoxide anion.

Although DMF and MeCN were shown to be excellent solvents for the reaction (Table 1, entries 3–6, 10, 15–23, and 26) we encountered difficulties in efficiently extracting the product upon quenching the reactions (1 M HCl). EtOAc was promising and made extraction very easy but during the reactions small quantities of a dark red, sticky, precipitate were observed (Table 1, entry 12). This was considered problematic for processing in a meso flow reactor due to the potential for causing blockages. It was however found that the addition of between 10–20% v/v MeCN ensure a fully homogeneous solution and also allowed for simple aqueous extraction with good recovery (>90%). However, from the provisional results DCM stood out as the most viable solvent (Table 1, entries 7, 24 and 25) and allowed a 0.25 M solution of substrate **9** to be processed with TMG or DBU (2.5 equiv) and ethyl cyanoacetate (**10**, 1.1 equiv) at 50 °C in quantitative conversion.

**Scheme 2:**
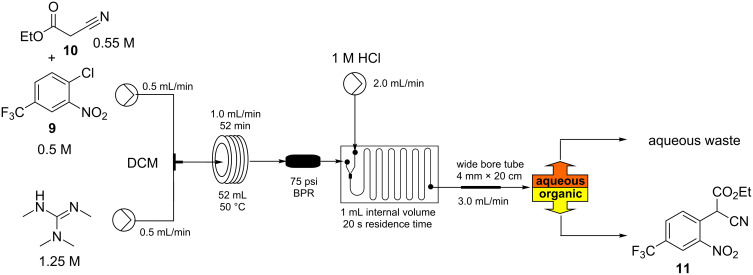
Initial flow reactor setup for the synthesis of intermediate **11**.

Based upon these screening results we devised a simple flow set-up where two stock solutions were united at a simple T-mixing piece and subsequently directed into a heated flow coil reactor maintained at 50 °C (Scheme 2). The intensely red coloured solution (anion of the S_N_Ar adduct) [8] which quickly formed was quenched after the incubation period (35–108 min) using a third flow stream of hydrochloric acid (1 M) blended via a dedicated mixer chip before the combined mixture was phase separated.

To enable the phase separation we utilised a passive membrane system based upon a modified Biotage universal separator [9]. This enabled the heavier chlorinated phase to be removed from the lower connection and for the lighter aqueous phase to be decanted from an overflow positioned run-off. At flow rates of 0.5–1.2 mL/min emanating from the main reactor this unit performed reliably giving excellent quenching and separation. However, at higher flow rates issues were encountered with incomplete partitioning (some emulsion formation) of the biphasic mixture resulting in the loss of product containing material to the aqueous run off. This was determined to be a result of the high shear generated in the in-line mixing chip at the higher flow rates and the need for longer settling times of the heavily segmented flow. This issue could be overcome by directing the aqueous run off from the first separator into a second equivalent unit or by simply splitting the original quenched flow over two parallel separators. Although functional these approaches were not the optimum in design or utilisation of the separator components. We therefore also investigated the replacement of the problematic mixing chip with various configured T- and Y-connectors but this immediately gave other issues due to incomplete quenching which resulted in poor product recovery and associated contamination. A more straightforward approach proved to be to introduce a flow stratification zone prior to the separator which was achieved through the expedient introduction of a section of wider bore tubing (expanded from the reactor i.d. 1.6 mm → 4.0 mm × 20 cm) [10]. This enabled reactor throughput flow rates of up to 2.0 mL (for flow rates above 1.0 mL a second 52 mL flow coil was added to the system) to be successfully quenched inline and then successively handled by a single Biotage universal separator. In all cases the output from the main reactor showed quantitative conversion with only the product being isolated following solvent evaporation. At a flow rate of 1.8 mL/min from the reactor this gave a maximum throughput of 27 mmol/h operating at steady state.

Despite the versatility of the reactor its productivity was lower than we ideally wanted and unfortunately DCM proved to be an incompatible solvent with the following hydrogenation step. Although solvent swapping would have been possible we determined that when EtOAc was used as the solvent and diluted with EtOH in the presence of acetic acid as an additive this allowed for the successful reductive cyclisation to the indole. As a result we investigated the scaled synthesis of intermediate **11** in EtOAc. Having had previous success with handling slurries in flow using the Coflore, AM technology ACR device [11], we decided to utilise this equipment in the synthesis to overcome the issues encountered with solubility [12–14].

**Scheme 3:**
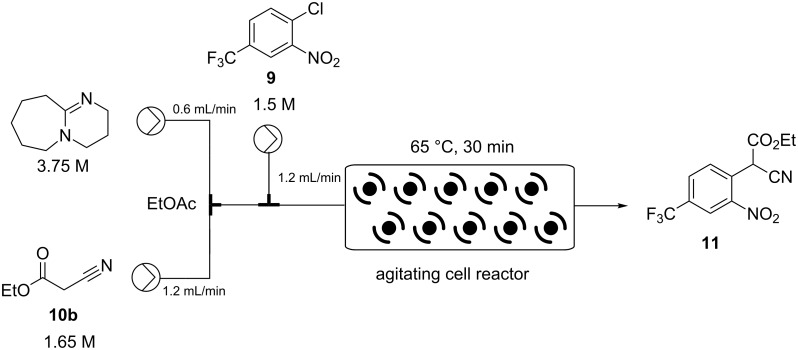
Coflore ACR setup for the synthesis of intermediate **11**.

The starting materials were prepared as individual stock solutions in EtOAc and mixed sequentially, first the base and the ethyl cyanoacetate (**10**) being combined (note this was an exothermic process) before meeting a solution of the aryl chloride **9** and entering the ACR which was agitated at 8 Hz (Scheme 3).

**Scheme 4:**
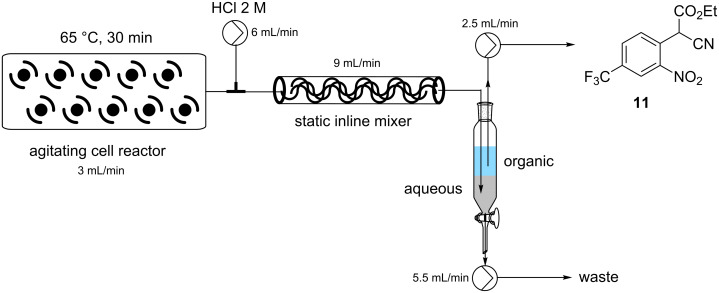
Quenching and work-up of the reaction stream from the Coflore ACR for the synthesis intermediate **11**.

In an attempt to intensify the process and with the specific aim of reducing the amount of solvent in order to maintain a viable working concentration in the subsequent reduction step (after dilution with EtOH) we undertook a series of concentration and flow rate optimisations. It was ultimately found that at a combined flow rate of 3 mL which equated to a residence time of approximately 30 min and a reactor temperature of 65 °C we were able to achieve a quantitative conversion of a 1.5 M solution of substrate **9**. Under continuous operation the reactor output was a dark red suspension comprising approx. 10% solid by volume but was easily processed through the system even over extended periods of time (>14 h). This set-up gave a theoretical working throughput of 0.108 mol/h. Next, to facilitate the integrated quenching and work-up we added a static mixing element [15] at the confluence point of an aqueous solution of 2 M HCl delivered at a flow rate of 6 mL/min (Scheme 4). Evidence for effective quenching was immediately observed by the transformation of the dark red reaction mixture to a pale yellow biphasic solution which quickly phase separated upon collection. Confirmation of the successful quenching was obtained by ^1^H NMR and LC analysis of the organic phase which indicated only the product **11** and trace amounts of ethyl cyanoacetate (**10**). Finally, manual separation followed by drying over Na_2_SO_4_ and solvent evaporation gave the desired product **11** in 94–96% yield, based upon 6 sampled aliquots of 20 min each processing time.

Several laboratory approaches to the automation of batch separation have been reported using machine vision systems [16–17], and inline detection devices employing optics [18] or inductive conductivity (impedance measurements) [19] to determine phase partitions. However, due to specific project time limitations we were constrained to use a more manual approach but would certainly incorporate such a labour saving device into a future development version. Our basic system used a simple batch collection vessel with two HPLC pumps with appropriately positioned inputs to remove independently the aqueous and organic phases. Occasional manual intervention allowed intermittent recalibration of the pumps to maintain working volumes and create a suitable phase segment for removal. Again, following evaporation of the solvent a high isolated yield of the target molecule **11** was attained in 93–97%.

**Figure 2:**
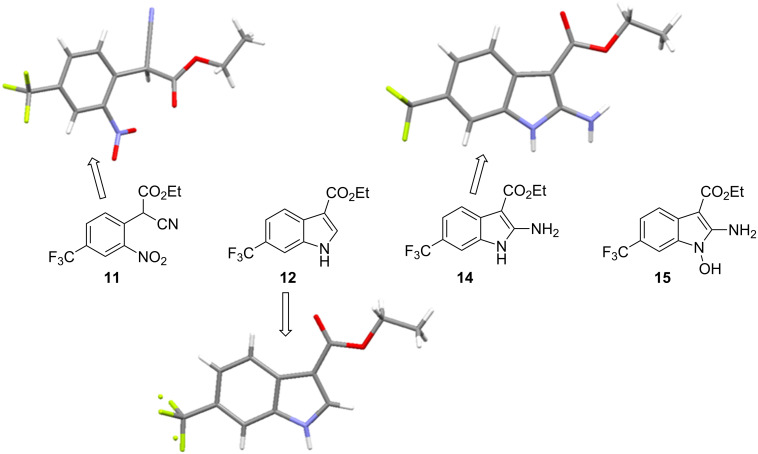
X-ray structure of intermediate **11**, and reductive cyclisation products **12** and **14**, assigned structure of byproduct **15**.

**Scheme 5:**
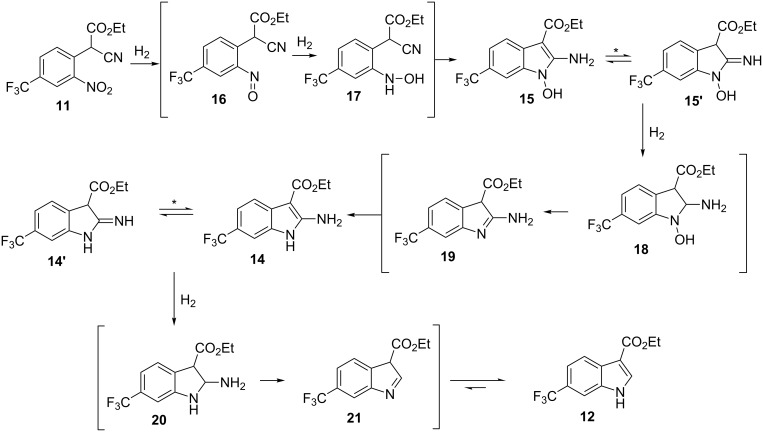
Stepwise reduction of intermediate **11** under hydrogenation conditions. * Indicates potential tautomeric structure. Species in parentheses are proposed transient intermediates.

The collected solution generated following aqueous quenching and separation contained 0.4 M product **11** which was further diluted with EtOH to furnish a 0.2 M stock solution for use in the next reductive cyclisation step. The reduction was performed using a ThalesNano H-cube system [20–21] operating in full hydrogen mode with a 10 mol % Pd/C catalyst cartridge. At a flow rate of 0.4 mL/min and a temperature of 50 °C full conversion to the indole product **12** was achieved (Table 2, entry 1). When the flow rate was raised to 0.8–1 mL/min complete consumption of the starting material was observed but a mixture of products was isolated (Table 2, entries 2 and 3). Careful analysis of the composition indicated the presence of the desired product (**12**, 62%) along with two other compounds (Figure 2), which following chromatographic isolation, were assigned as the amino indole (**14**, 29%) and the *N*-hydroxyamino indole (**15**, 9%). As expected these latter two products resulting from incomplete reduction were obtained in greater amounts as the flow rate was further increased along with an increasing proportion of the hydroxyaminoindole **15**. This correlates with previous literature findings employing catalysed hydrogenation [22–26] or stoichiometric metal (zinc or indium) mediated reduction and is consistent with a stepwise reduction mechanism (Scheme 5) [27–29]. It is evident from the sequence that several equivalents of hydrogen are necessary for full reduction and as a result a higher concentration (pressure) of hydrogen would be beneficial (Table 2, entries 4–10). We also noted that according to the mechanism protonation of the anilino nitrogen could be beneficial in promoting the reduction steps as well as aiding in the loss of water (**16** and **18**) and ammonia (**20**). We therefore screened a series of acid catalysts which highlighted acetic acid (10–30 mol %) as the optimum additive (Table 2, entries 11–14). Stronger acids or higher loading of acid often resulted in the generation of high internal pressures and in certain cases the formation of precipitates was noted requiring premature termination of the run and a safe shutdown. Using acetic acid and limiting the acid concentration (10 mol %) whilst working at a higher internal pressure (15 bar) enabled stable and continuous operation permitting the flow rate to be raised to 1.3 mL/min whilst ensuring a >98% conversion to the desired indole **12** (Table 2, entries 15–18) [30–31]. This translated into a throughput of 15.6 mmol/h (3.7 g/h product). The final product could be easily isolated in 93% yield as an off white solid by solvent evaporation and trituration with 9:1 hexane/Et_2_O. This purification removed both residual acetic acid and, if present, small traces of byproducts.

**Table 2:** Selected optimisation experiments for reductive cyclisation to compound **12**.^a^

Entry	AcOH (mol %)	Temp. (°C)	Pressure (bar)	Flow rate (mL/min)	Product composition ratio **11**:**12**:**14**:**15**

1	0	50	0	0.4	0:100:0:0
2	0	50	0	0.8	0:62:29:9
3	0	50	0	1.0	10:30:40:20
4	0	50	5	0.8	0:86:11:3
5	0	50	10	0.8	0:95:5:0
6	0	50	15	0.8	0:99:1:0
7	0	50	15	1.2	0:77:18:5
8	0	40	5	0.8	4:43:19:34
9	0	60	5	0.8	0:83:12:5
10	0	70	5	0.8	3:46:23:9^b^
11	5	50	0	0.8	0:80:17:3
12	10	50	0	0.8	0:93:6:1
13	20	50	0	0.8	0:95:4:1
14	50	50	0	0.8	0:94:5:0
15	10	50	15	1.1	0:99:1:0
16	10	50	15	1.2	0:100:0:0
17	10	50	15	1.3	0:98:2:0
18	10	50	15	1.4	0:89:8:3

^a^Reaction concentration of **11** was 0.2 M in EtOH/EtOAc 50:50 v/v. Composition analysis was performed by LC–MS against isolated standards. ^b^An additional product of almost double the mass of **12** was observed in the LC–MS but this material could not be isolated. The catalyst cartridge rapidly lost activity and could not be regenerated through washing.

In the next sequence we looked at telescoping the final two steps of the process; substitution of the ethoxy group by hydrazine and then ring formation to the 3*H*-[1,3,4]oxadiazol-2-one unit [32–33].

**Scheme 6:**
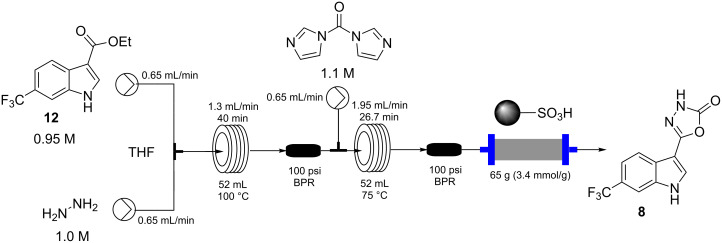
Flow sequence for the construction of product **8**.

In this procedure a 0.95 M THF solution of compound **12** was united with a solution of hydrazine (1.0 M in THF) and directed into a heated flow coil to be superheated at 100 °C (Scheme 6). A residence time of 40 min allowed full conversion to the corresponding acyl hydrazine **13** which was directly intercepted with a further input stream containing CDI (1.1 M in THF) and heated at 75 °C for an additional 26 min in a second flow coil. This furnished the final product **8** in quantitative conversion with the product stream being readily purified by passage through a scavenging cartridge of QP-SA (a sulfonic acid functionalised polymer). The product **8** was obtained after solvent evaporation as a yellow solid (94%) but required recrystallization from DCM to give a white amorphous powder of high purity in 82% yield.

A series of attempts to employ dimethyl carbonate as a replacement reagent for CDI in the final step failed under a range of conditions as did trials to directly utilise (methoxycarbonyl)hydrazide (CAS 6294-89-9) in the previous acyl hydrazine forming step. However, we found that triphosgene (0.4 equiv) could be successfully used in the latter cyclisation process [34–37]. Using a similar reactor assembly as per Scheme 6 the triphosgene (0.8 M in CHCl_3_) was combined with the solution of intermediate **13** and passed through a heated (55 °C) flow coil and then through a packed bed scavenging cartridge containing QP-DMA (*N*,*N*-dimethylbenzylamine polystyrene). Following solvent evaporation this gave the product as an off white solid in 91% isolated yield. In practice this approach proved far superior to the previously employed CDI cyclisation providing higher overall yields and improved quality product direct from the reactor. Indeed, the final product **8** was of sufficient purity for direct application allowing on demand and scalable production from a stable intermediate **12** in less than 2 h from reactor start-up.

## Conclusion

**Scheme 7:**
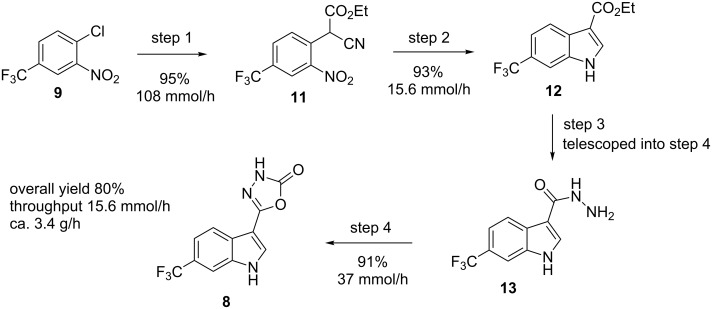
Assembled process for flow synthesis of product **8** with yields and throughputs.

Using the procedures outlined we have been able to rapidly assembly the desired target molecule **8** through a flexible workflow generating sufficient material for on-going field trials. Obviously in the context of our currently assembled route the reductive cyclisation to the indole unit **12** is the process limiting step (Scheme 7). However, several options including commercially available larger scale flow apparatus for performing such flow hydrogenations are available (i.e., H-Cube Mid [38], FlowCAT [39]) [40–41]. However, as intermediate **12** was shown to be a highly stable structure, in practice, this created a convenient staging point to generate intermediate holding batches of material for subsequent on demand processing. Overall we envisage this laboratory scale design to offer several opportunities for further development enabling quick up regulation of production in the future.

## Experimental

### Material and methods

Unless otherwise stated, all solvents, substrates and reagents were used as purchased without further purification.

^1^H NMR spectra were recorded on Bruker Avance-400 instruments and are reported relative to residual solvent: CHCl_3_ (δ 7.26 ppm), DMSO (δ 2.50 ppm). ^13^C NMR spectra were recorded on the same instruments and are reported relative to CHCl_3_ (δ 77.16 ppm) or DMSO (δ 39.52 ppm). Data for ^1^H NMR are reported as follows: chemical shift (δ ppm) (multiplicity, coupling constant (Hz), integration). Multiplicities are reported as follows: s = singlet, d = doublet, t = triplet, q = quartet, p = pentet, m = multiplet, br. s = broad singlet, app = apparent. Data for ^13^C NMR are reported in terms of chemical shift (δ ppm) and multiplicity (C, CH, CH_2_ or CH_3_). Data for ^19^F NMR were recorded on the above instruments at a frequency of 376 MHz using CFCl_3_ as external standard. For all compounds DEPT-135, COSY, HSQC, and HMBC were run and used in the structural assignment. IR spectra were recorded neat (ATR sampling) with the intensities of the characteristic signals being reported as weak (w, <20% of tallest signal), medium (m, 20–70% of tallest signal) or strong (s, >70% of tallest signal). Low and high-resolution mass spectrometry was performed using the indicated techniques on instruments equipped with Acquity UPLC and a lock-mass electrospray ion source. For accurate mass measurements the deviation from the calculated formula is reported in ppm. Melting points were recorded on an automated melting point system with a heating rate of 1 °C/min and are uncorrected. Microwave optimisation reactions were performed in a Biotage^®^ Initiator+ microwave system.

Flow reactions were preformed using the pumping system of a Vapourtec R-Series modular flow chemistry system [42]. Heating for the 52 mL flow coils was provided using a Polar Bear Plus unit [43].

**Ethyl 2-cyano-2-(2-nitro-4-(trifluoromethyl)phenyl)acetate (11)** [44]**:**


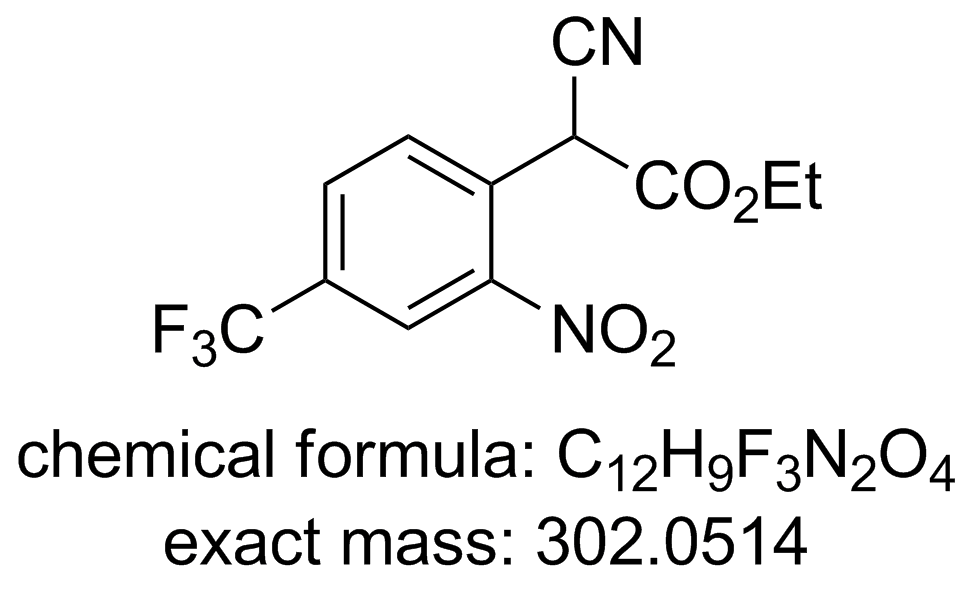


**Process 1 (DCM):** Two stock solutions in DCM were prepared. Solution A: TMG (1.25 M, 2.5 equiv) and solution B: a mixture of 4-chloro-3-nitrobenzotrifluoride (0.5 M, 1.0 equiv) and ethyl cyanoacetate (0.55 M, 1.1 equiv). The two stock solutions were pumped (0.65 mL/min each channel) direct from their respective reservoirs to combine at a PEEK T-piece and were then directed through a FEP flow coil (52 mL maintained at 50 °C using a Polar Bear Plus reactor – Cambridge Reactor Design). The system pressure was controlled using a 75 psi inline back pressure regulator. The flow stream was quenched by mixing with a solution of hydrochloric acid (1 M) at a flow rate of 2 mL/min within a glass microreactor (1 mL, Little Things Factory). The biphasic mixture was passed into a section of wide bore FEP tubing (4 mm i.d × 20 cm length) and on into a modified Biotage Universal Separator (Biotage) allowing separation of the organic and aqueous fluidic flows. The organic layer was dried (Na_2_SO_4_) and the solvent evaporated to yield the title compound in 94–96%.

**Process 2 (EtOAc):** Three EtOAc stock solutions were prepared. Solution A: DBU (3.75 M, 2.5 equiv), solution B: ethyl cyanoacetate (1.65 M, 1.1 equiv), and solution C: 4-chloro-3-nitrobenzotrifluoride (1.5 M, 1.0 equiv). Stock solutions A (0.6 mL/min) and B (1.2 mL/min) were pumped from their respective reservoirs and combined at a PEEK T-piece. The combined flow was then combined at a second PEEK T-piece with solution C (1.2 mL/min) and the flow directed into the Coflore ACR (spring inserts). The ACR was agitated at 8 Hz and heated at 65 °C. The flow stream was quenched by mixing with a solution of hydrochloric acid (2 M) at a flow rate of 6 mL/min using a inline static mixing element (Esska [15]). The biphasic mixture was passed into a collection vessel. The input lines for two Knauer K120 HPLC pumps were positioned to draw independently the aqueous and organic phases. The organic layer was collected and dried (Na_2_SO_4_) and the solvent evaporated to yield the title compound. The product was isolated in 93–97% yield.

^1^H NMR (400 MHz, chloroform-*d*) δ 8.51 (dt, *J* = 2.0, 0.6 Hz, 1H), 8.12–7.86 (m, 2H), 5.79 (s, 1H), 4.35 (q, *J* = 7.2 Hz, 2H), 1.37 (t, *J* = 7.2 Hz, 3H); ^13^C NMR (101 MHz, CDCl_3_) δ 162.8 (C), 147.6 (C), 133.3 (q, *J* = 35 Hz, C), 132.4 (CH), 131.0 (q, *J* = 4 Hz, CH), 128.9 (C), 123.7 (q, *J* = 4 Hz, CH), 122.2 (q, *J* = 273 Hz, C), 113.8 (C), 64.4 (CH_2_), 41.2 (CH), 13.9 (CH_3_); ^19^F NMR (376 MHz, CDCl_3_) δ −63.2; IR (neat) ν/cm^−1^: 3092 (w), 2925 (w), 1732 (m), 1537 (m), 1502 (m), 1357 (m), 1324 (s), 1257 (s), 1182 (s), 1138 (s), 1090 (s), 1012 (m), 866 (m), 825 (m), 698 (m); LC–MS (TOF^+^) 302.1 (M + H); HRMS (ESI) *m*/*z*: calcd for C_12_H_8_N_2_O_4_F_3_, 301.0436; found, 301.0452 (Δ = 5.3 ppm); melting range: 60.5–61.6 °C; X-ray crystal data: CCDC 1572810.

**Ethyl 6-(trifluoromethyl)-1*H*-indole-3-carboxylate (12)** [23]**:**


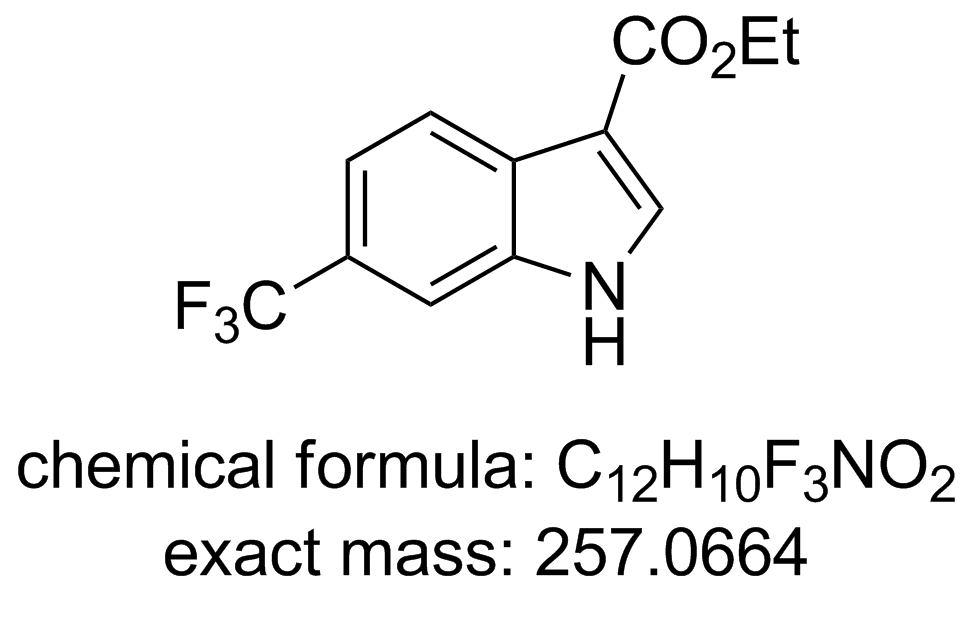


A 0.2 M solution of compound **11** in a 1:1 mixture of EtOAc/EtOH with 10 mol % AcOH was passed through a ThalesNano H-cube at 1.3 mL/min containing a 10 mol % Pd/C heated at 50 °C and pressurised at 15 bar. The solvent was removed under reduced pressure and the residue triturated with 9:1 hexane/Et_2_O. The product was isolated in 93% yield. ^1^H NMR (400 MHz, DMSO-*d*_6_) δ 12.33 (s, 1H), 8.31 (d, *J* = 3.0 Hz, 1H), 8.18 (dd, *J* = 8.5, 0.9 Hz, 1H), 7.83 (dt, *J* = 1.7, 0.9 Hz, 1H), 7.49 (dd, *J* = 8.5, 1.7 Hz, 1H), 4.31 (q, *J* = 7.1 Hz, 2H), 1.34 (t, *J* = 7.1 Hz, 3H); ^13^C NMR (101 MHz, DMSO-*d*_6_) δ 164.4 (C), 135.8 (d, *J* = 3 Hz, CH), 128.7 (C), 125.4 (q, *J* = 273 Hz, C), 123.3 (q, *J* = 34 Hz, C), 121.75 (CH), 118.0 (q, *J* = 4 Hz, CH), 117.6 (C), 110.3 (q, *J* = 4 Hz, CH), 107.5 (C), 59.8 (CH_2_), 14.9 (CH_3_); ^19^F NMR (376 MHz, DMSO-*d*_6_) δ −59.3; IR (neat) ν/cm^−1^: 3196 (m), 1667 (s), 1514 (m), 1440 (m), 1328 (s), 1228 (s), 1191 (m), 1160 (s), 1117 (s), 1055 (s), 826 (m), 745 (m), 674 (m), 615 (m), 524 (m); LC–MS (TOF^+^) 258.1 (M + H); HRMS (ESI) *m*/*z*: calcd for C_12_H_11_NO_2_F_3_, 258.0742; found, 258.0753 (Δ = 4.3 ppm); melting range: 197.3–200.0 °C; X-ray crystal data: CCDC 1572811.

**Ethyl 2-amino-6-(trifluoromethyl)-1*H*-indole-3-carboxylate (14)** [45]**:**


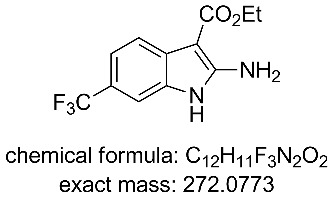


^1^H NMR (400 MHz, DMSO-*d*_6_) δ 10.90 (br s, 1H), 7.68 (d, *J* = 8.0 Hz, 1H), 7.44 (s, 1H), 7.27 (d, *J* = 8.0 Hz, 1H), 7.00 (s, 2H), 4.26 (q, *J* = 7.2 Hz, 2H), 1.33 (t, *J* = 7.2 Hz, 3H); ^13^C NMR (101 MHz, DMSO-*d*_6_) δ 165.9 (C), 155.3 (C), 132.5 (C), 130.5 (C), 126.0 (CF_3_, q, *J* = 271 Hz), 119.8 (C), 118.1 (CH, q, *J* = 31 Hz), 117.6 (CH, q, *J* = 4 Hz), 106.9 (CH, q, *J* = 4 Hz), 84.5 (C), 58.8 (CH_2_), 15.1 (CH_3_); ^19^F NMR (376 MHz, DMSO-*d*_6_) δ −58.6; IR (neat) ν/cm^−1^: 3496 (m), 3344 (m), 1645 (m), 1619 (s), 1555 (m), 1506 (s), 1379 (m), 1325 (s), 1230 (m), 1152 (s), 1099 (s), 1070 (s), 1052 (s), 856 (m), 812 (s), 671 (m), 530 (m); LC–MS (TOF^+^) 273.1 (M + H); HRMS (ESI) *m*/*z*: calcd for C_12_H_12_N_2_O_2_F_3_, 273.0851; found, 273.0860 (Δ = 3.3 ppm); melting range: >140 °C (decomposition); X-ray crystal data: CCDC 1572812.

**Ethyl 2-amino-1-hydroxy-6-(trifluoromethyl)-1*H*-indole-3-carboxylate (15)** [25]**:**


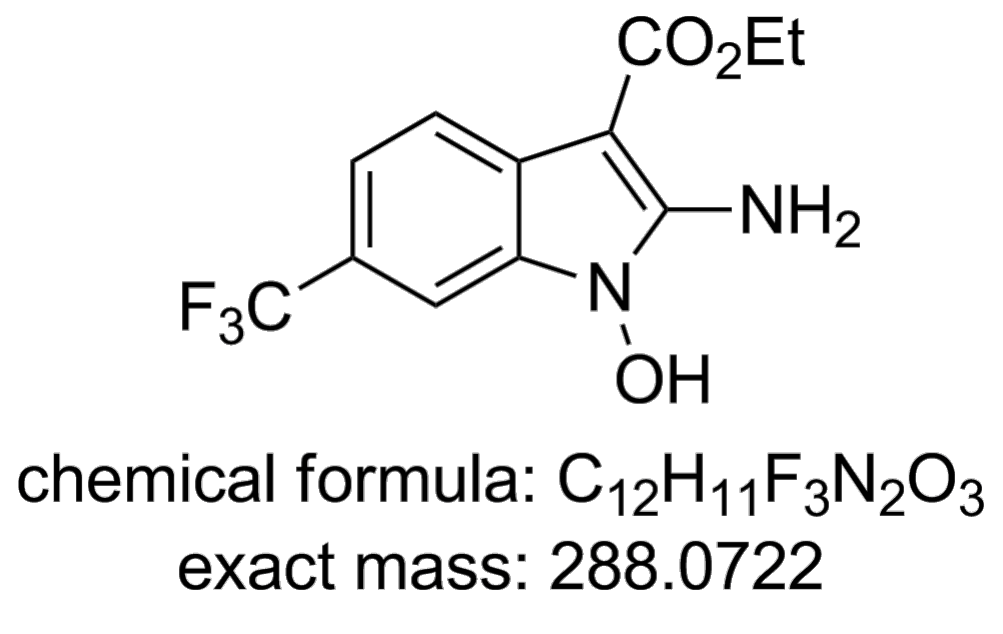


^1^H NMR (400 MHz, DMSO-*d*_6_) δ 11.55 (br s, 1H), 7.75 (d, *J* = 8.2 Hz, 1H), 7.36 (s, 1H), 7.33 (d, *J* = 8.2 Hz, 1H), 4.26 (q, *J* = 7.2 Hz, 2H), 1.33 (t, *J* = 7.2 Hz, 3H); ^13^C NMR (101 MHz, DMSO-*d*_6_) δ 165.3 (C), 151.9 (C), 131.5 (C), 125.8 (CF_3_, q, *J* = 272 Hz), 125.7 (C), 120.0 (C, q, *J* = 32 Hz), 118.4 (CH), 118.1 (CH, q, *J* = 2 Hz), 103.7 (CH, q, *J* = 4 Hz), 80.4 (C), 58.9 (CH_2_), 15.2 (CH_3_); ^19^F NMR (376 MHz, DMSO-*d*_6_) δ −58.7; IR (neat) ν/cm^−1^: 3266 (br), 3113 (br), 2981 (m), 1748 (s), 1696 (s), 1632 (m), 1441 (s), 1323 (s), 1254 (m), 1223 (m), 1172 (s), 1120 (s), 1060 (s), 879 (m), 824 (m), 668 (m); LC–MS (TOF^+^) 289.1 (M + H); HRMS (ESI) *m*/*z*: calcd for C_12_H_12_N_2_O_3_F_3_, 289.0800; found, 289.0805 (Δ = 1.7 ppm); melting range: decomposition >145 °C.

**6-(Trifluoromethyl)-1*H*-indole-3-carbohydrazide (13):**


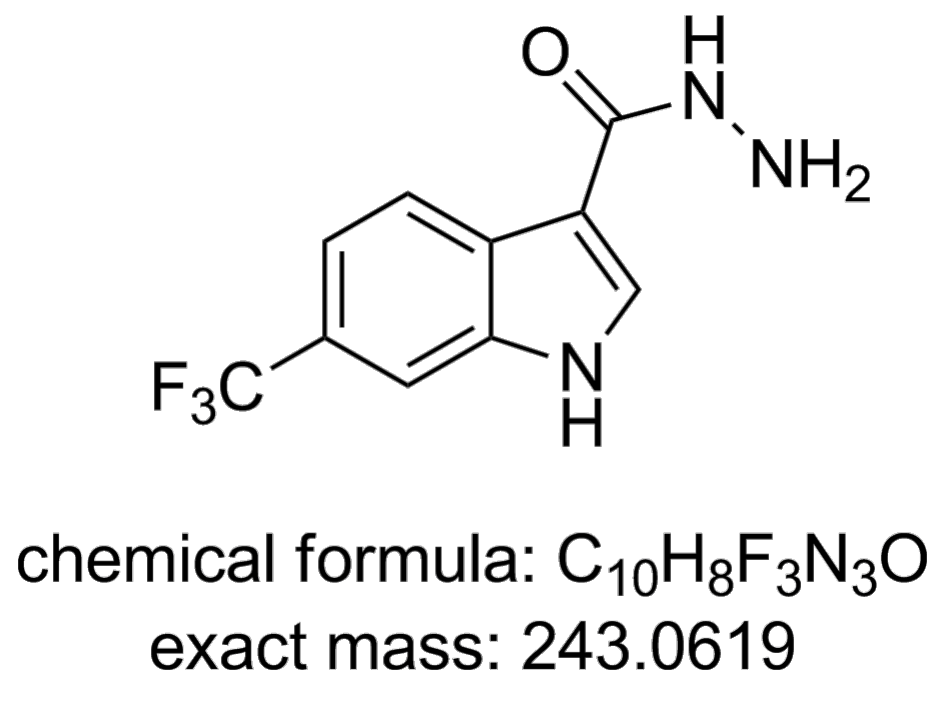


Two stock solutions in THF were prepared. Solution A: hydrazine (1.00 M, 1.05 equiv) and solution B containing compound **11** (0.95 M, 1.0 equiv). The two stock solutions were pumped (0.65 mL/min each channel) from their respective reservoirs to combine at a PEEK T-piece and were then directed through a FEP flow coil (52 mL maintained at 100 °C using a Polar Bear Plus reactor – Cambridge Reactor Design, residence time 40 min). A 100 psi inline back pressure regulator was positioned prior to the exit. Collection of the reactor output allowed isolation of the title compound in 81% isolated yield following column silica chromatography (DCM/MeOH 9:1). ^1^H NMR (400 MHz, DMSO-*d*_6_) δ 11.42 (br. s, 1H), 9.33 (s, 1H), 8.33 (d, *J* = 8.4 Hz, 1H), 8.19 (s, 1H), 7.89–7.70 (m, 1H), 7.41 (dd, *J* = 8.4, 1.7 Hz, 1H), 2.95 (br. s, 2 H); ^13^C NMR (101 MHz, DMSO-*d*_6_) δ 164.9 (C), 135.3 (C), 130.5 (CH), 129.2 (C), 125.6 (q, *J* = 272 Hz, C), 122.8 (q, *J* = 31 Hz, C), 122.2 (CH), 117.0 (q, *J* = 4 Hz, CH), 109.8 (C), 109.8 (q, *J* = 4 Hz, CH); ^19^F NMR (376 MHz, DMSO-*d*_6_) δ −59.0; IR (neat) ν/cm^−1^: 2900–3300 (broad), 3116 (m), 1615 (m), 1519 (m), 1376 (m), 1331 (s), 1238 (m), 1142 (m), 1096 (s), 1049 (s), 960 (m), 916 (m), 866 (s), 816 (s), 662 (s); LC–MS (TOF^+^) 244.1 (M + H); HRMS (ESI) *m*/*z*: calcd for C_10_H_9_N_3_OF_3_, 244.0698; found, 244.0700 (Δ = 0.8 ppm); melting range: >220 °C (decomposition).

**5-(6-(Trifluoromethyl)-1*H*-indol-3-yl)-1,3,4-oxadiazol-2(3*H*)-one (8):**


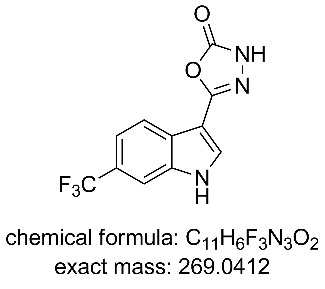


**Stage 1:** Two stock solutions in THF were prepared. Solution A: hydrazine (1.00 M, 1.05 equiv) and solution B containing compound **11** (0.95 M, 1.0 equiv). The two stock solutions were pumped (0.65 mL/min each channel) from their respective reservoirs to combine at a PEEK T-piece and were then directed through a FEP flow coil (52 mL maintained at 100 °C using a Polar Bear Plus reactor – Cambridge Reactor Design, residence time 40 min). A 100 psi inline back pressure regulator was added to control the system pressure.

**Stage 2 (CDI):** The reactor stream was further combined with a input of CDI (1.1 M, 1.1 equiv) in THF (0.65 mL/min). The unifided flow was directed through a second FEP flow coil (52 mL heated at 75 °C using a Polar Bear Plus reactor – Cambridge Reactor Design, residence time 26.7 min). A 75 psi inline back pressure regulator was positioned at the exit of the coil reactor to control the system pressure. A scavenging cartridge of QP-SA (65 g, 3.4 mmol/g loading) was placed in the flow path of the exiting solution. The reactor output was collected and the solvent was evaporated under reduced pressure allowing isolation of title compound **8** in 82% yield following recrystalisation from DCM.

**Alternative stage 2 (triphosgene):** The reactor stream was further combined with a input of triphosgene (0.8 M, 0.4 equiv) in THF (0.325 mL/min). The unified flow was directed through a second FEP flow coil (52 mL heated at 55 °C using a Polar Bear Plus reactor – Cambridge Reactor Design, residence time 32 min). A 75 psi inline back pressure regulator was positioned at the exit of the coil reactor to control the system pressure. A scavenging cartridge of QP-DMA (70 g, 2.4 mmol/g loading) was placed in the flow path of the exiting solution. The reactor output was collected and the solvent was evaporated under reduced pressure and enabling isolation of the title compound **8** in 91% yield as an off white solid.

^1^H NMR (400 MHz, DMSO-*d*_6_) δ 12.35 (br. s, 1H), 12.29 (br. s, 1H), 8.23 (d, *J* = 1.4 Hz, 1H), 8.09 (d, *J* = 8.4 Hz, 1H), 7.85 (m, 1H), 7.50 (dd, J = 8.4, 1.4 Hz, 1H); ^13^C NMR (101 MHz, DMSO-*d*_6_) δ 154.6 (C), 152.4 (C), 135.8 (C), 131.0 (CH), 126.8 (C), 125.3 (C, q, *J* = 272 Hz), 123.7 (C, q, *J* = 30 Hz), 121.4 (CH), 117.7 (CH, q, *J* = 4 Hz), 110.2 (CH, q, *J* = 5 Hz), 100.9 (C); ^19^F NMR (376 MHz, DMSO-*d*_6_) δ −59.3; IR (neat) ν/cm^−1^: 3344 (br), 2820 (br), 1750 (s), 1628 (s), 1507 (m), 1455 (m), 1332 (s), 1224 (m), 1160 (m), 1100 (s), 1052 (s), 977 (m), 915 (m), 920 (m), 751 (m), 738 (s), 619 (s);^.^ LC–MS (TOF^+^) 270.3 (M + H); HRMS (ESI) *m*/*z*: calcd for C_11_H_7_N_3_O_2_F_3_, 270.0490; found, 270.0497 (Δ = 2.6 ppm); melting range: decomposition >220 °C; X-ray crystal data: CCDC 1572809.

## Supporting Information

File 1 Reproductions of ^1^H and ^13^C NMR spectra for the reported compounds.
