# Supplementary material for: A concise flow synthesis of indole-3-carboxylic ester and its derivatisation to an auxin mimic
Source: Beilstein J Org Chem. 2017 Nov 29;13:2549–60. doi: 10.3762/bjoc.13.251 (PMC5727791; doi:10.3762/bjoc.13.251)
Supplement: File 1 — Reproductions of 1H and 13C NMR spectra for the reported compounds. [file Beilstein_J_Org_Chem-13-2549-s001.pdf]

Supporting Information

for

A concise flow synthesis of indole-3-carboxylic ester and its derivatisation to an auxin mimic

Marcus Baumann[1], Ian R. Baxendale*[1] and Fabien Deplante[1]

Address: [1]Department of Chemistry, University of Durham, South Road, Durham, Durham, DH1 3LE, UK

Email: Ian R. Baxendale - i.r.baxendale@durham.ac.uk

* Corresponding author

Reproductions of 1H and 13C NMR spectra for the reported compounds

Table of Contents:

1. Copies of NMR spectra S2

1. Copies of NMR spectra

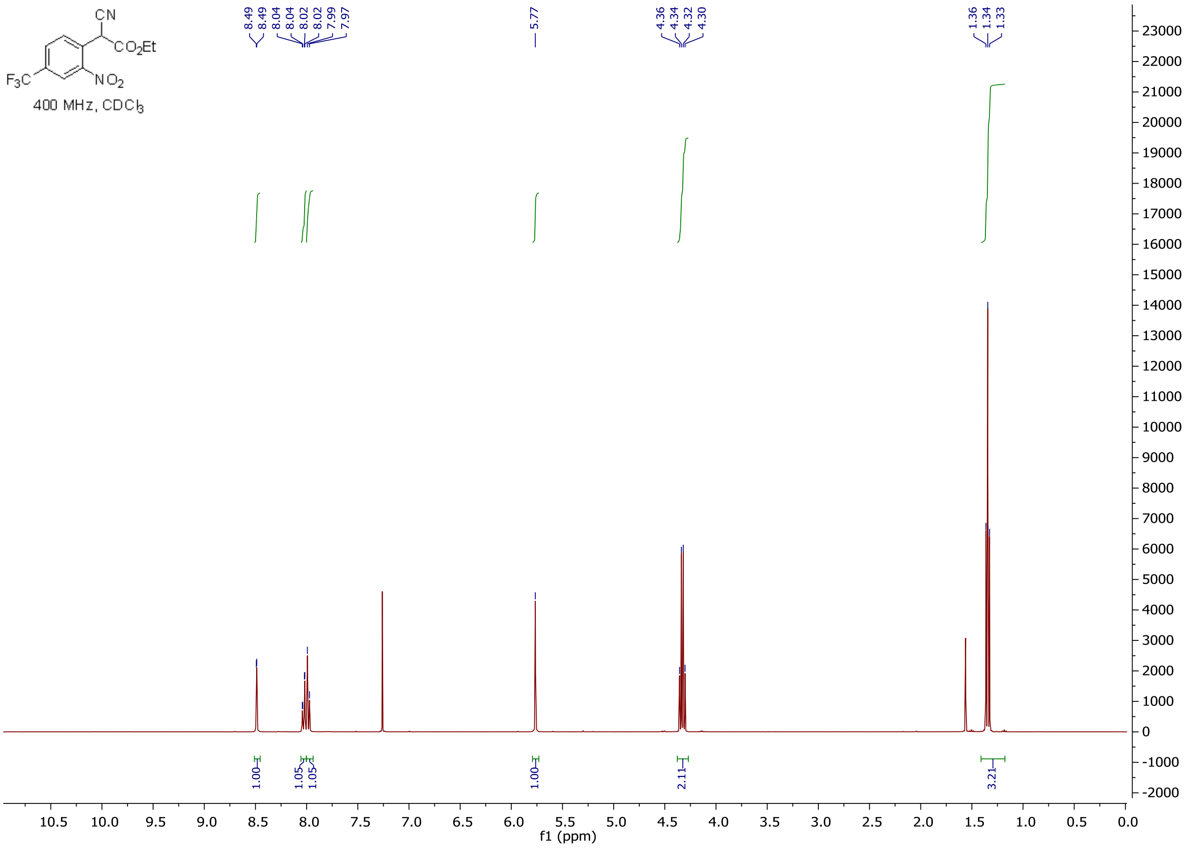

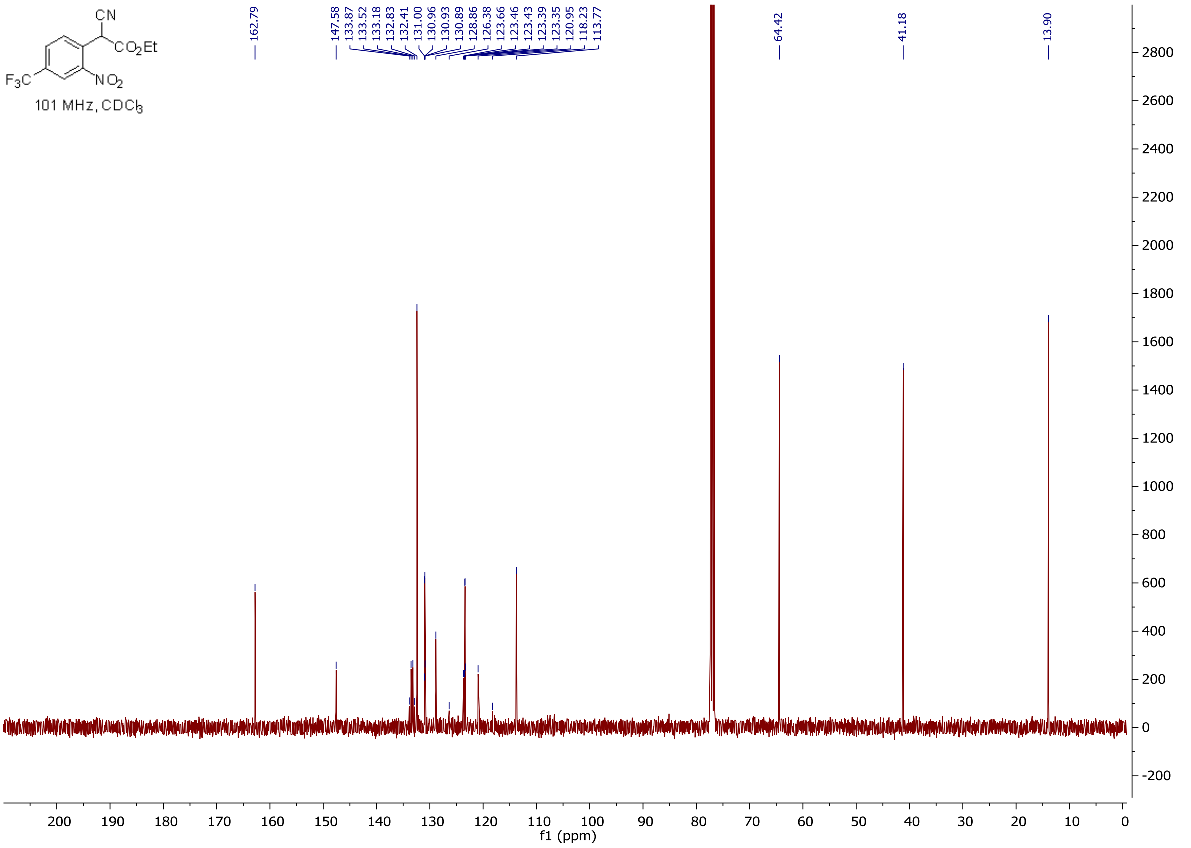

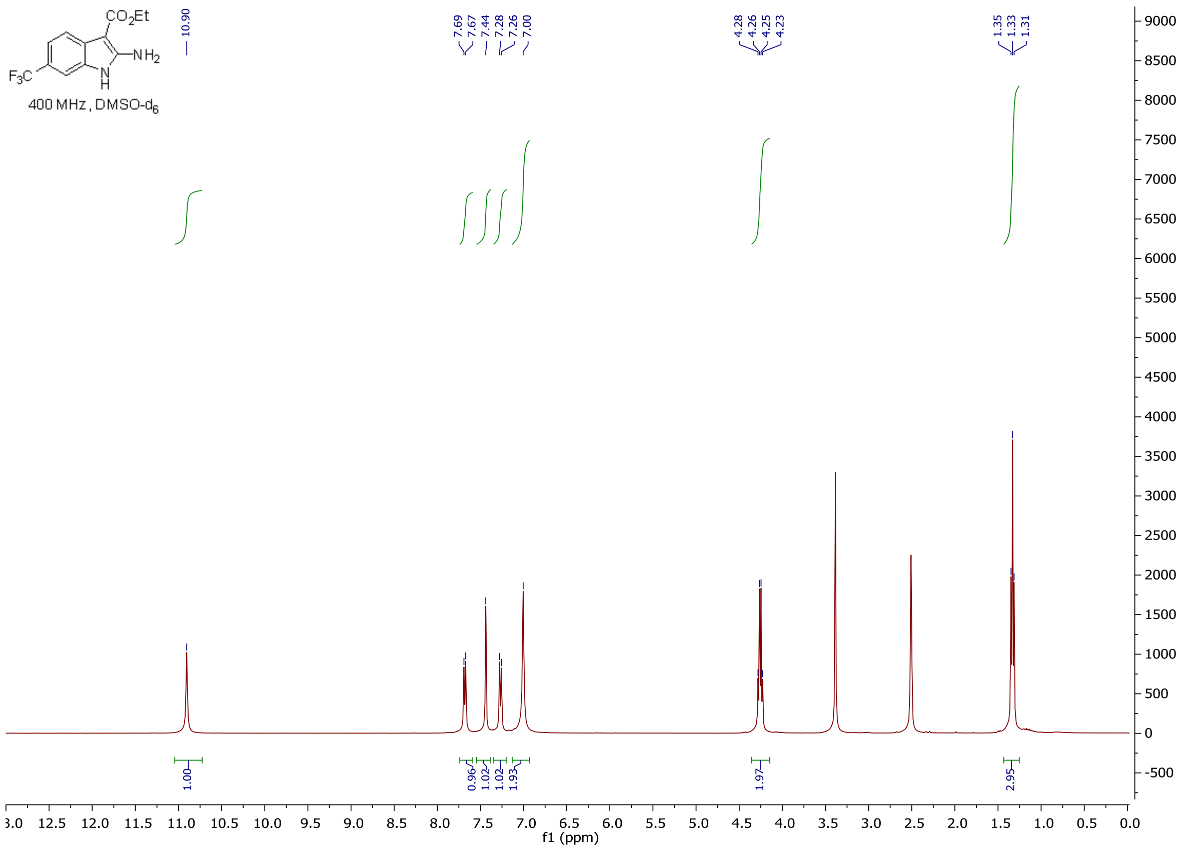

CO2Et
NH2
F3C
N
H
400 MHz, DMSO-d6
10.90
7.69
7.67
7.44
7.28
7.26
7.00
4.28
4.26
4.25
4.23
1.35
1.33
1.31
1.00
0.96
1.02
1.02
1.93
1.97
2.95
f1 (ppm)

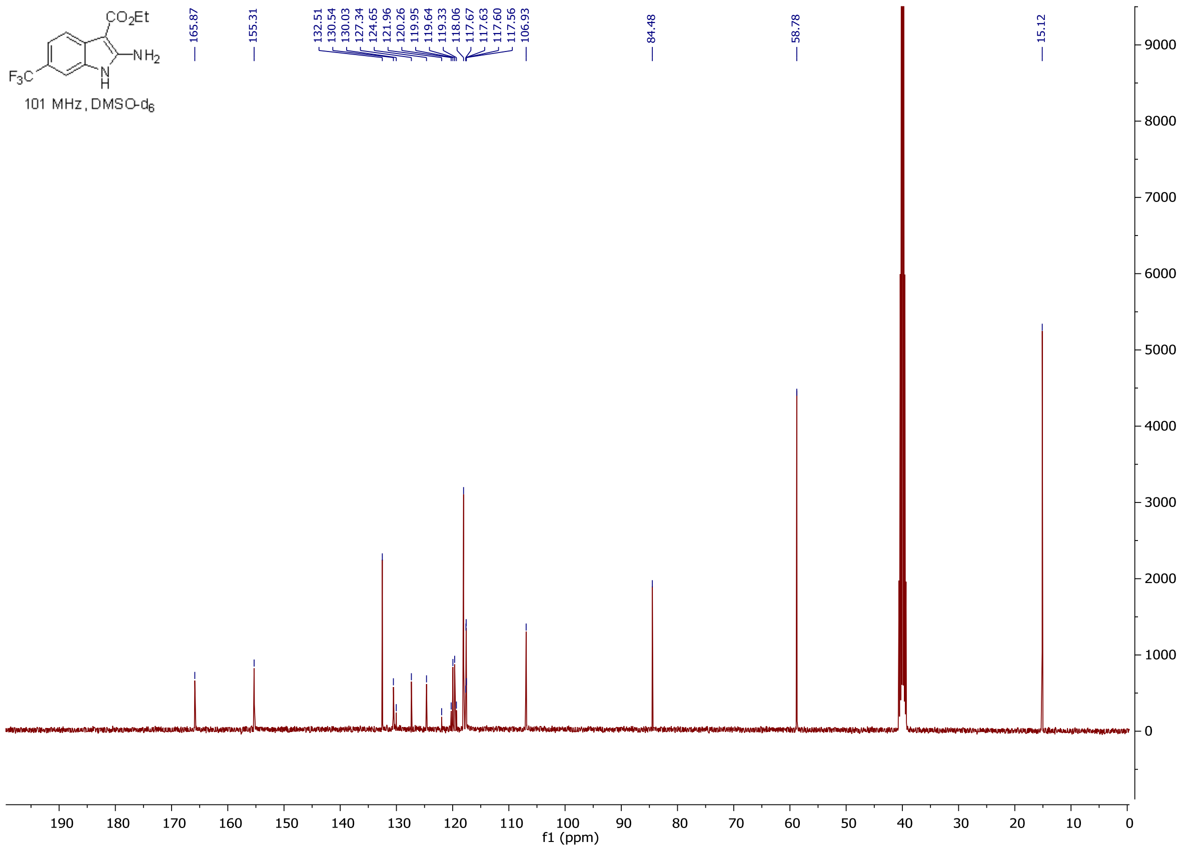

CO2Et
NH2
F3C
N
H
101 MHz, DMSO-d6
165.87
155.31
132.51
130.54
130.03
127.34
124.65
121.96
120.26
119.95
119.64
119.33
118.06
117.67
117.63
117.60
117.56
106.93
84.48
58.78
15.12
f1 (ppm)

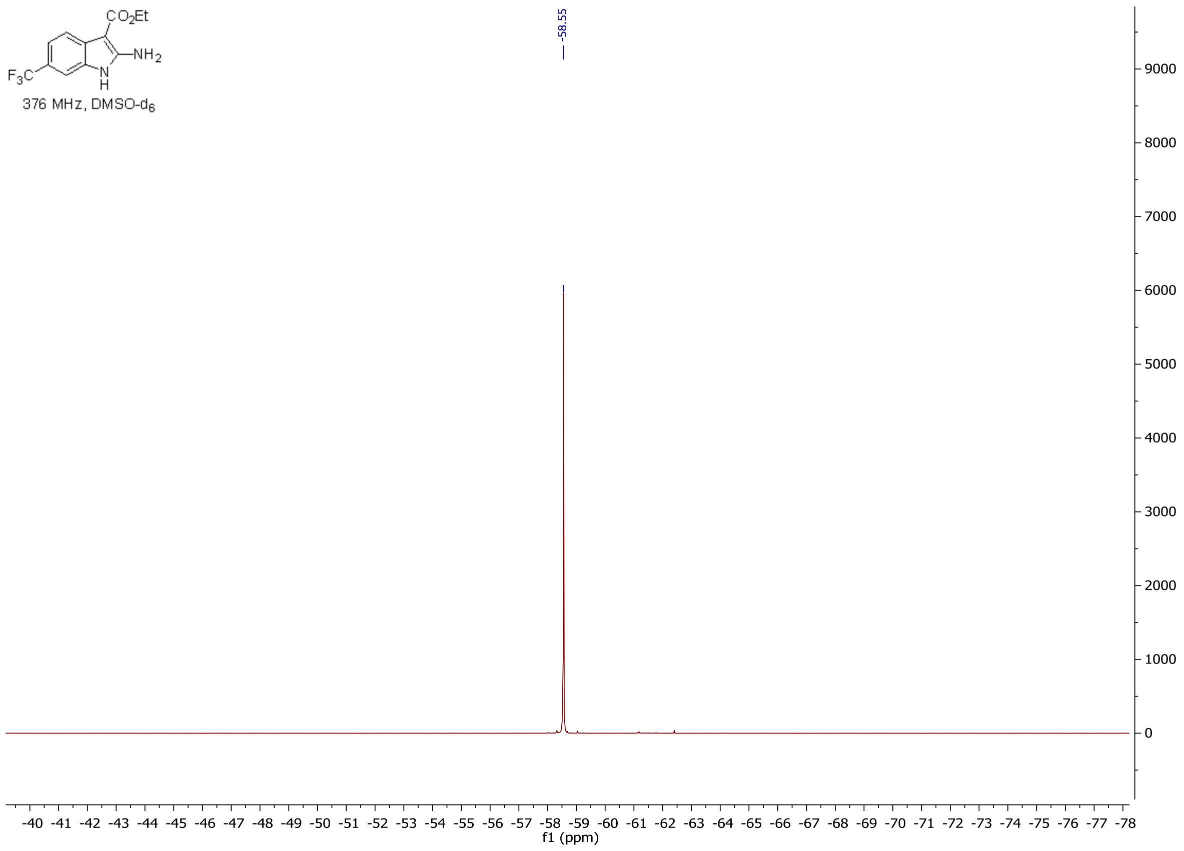
CO2Et
NH2
F3C
N
H
376 MHz, DMSO-d6
-58.55
f1 (ppm)

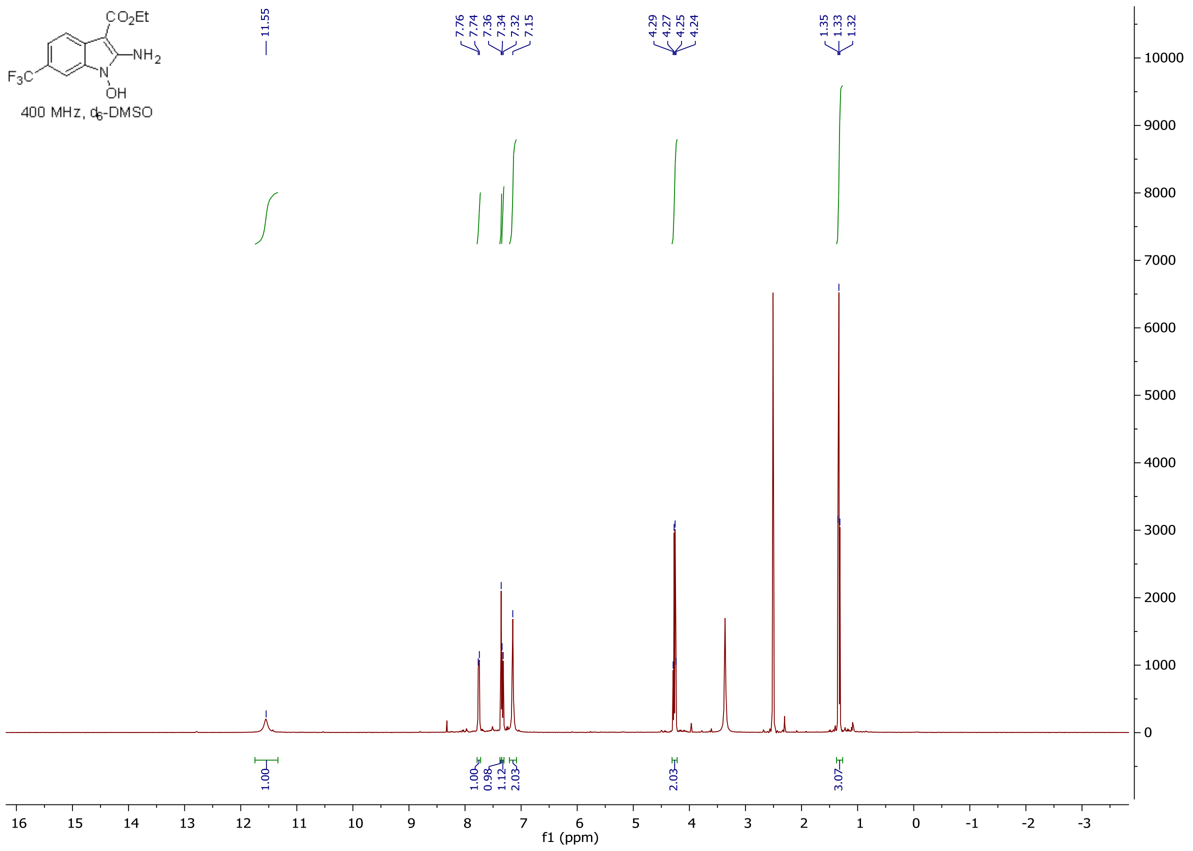
CO2Et
NH2
F3C
N
OH
400 MHz, d6-DMSO
11.55
7.76
7.74
7.36
7.34
7.32
7.15
4.29
4.27
4.25
4.24
1.35
1.33
1.32
1.00
1.00
0.98
1.12
2.03
2.03
3.07
f1 (ppm)

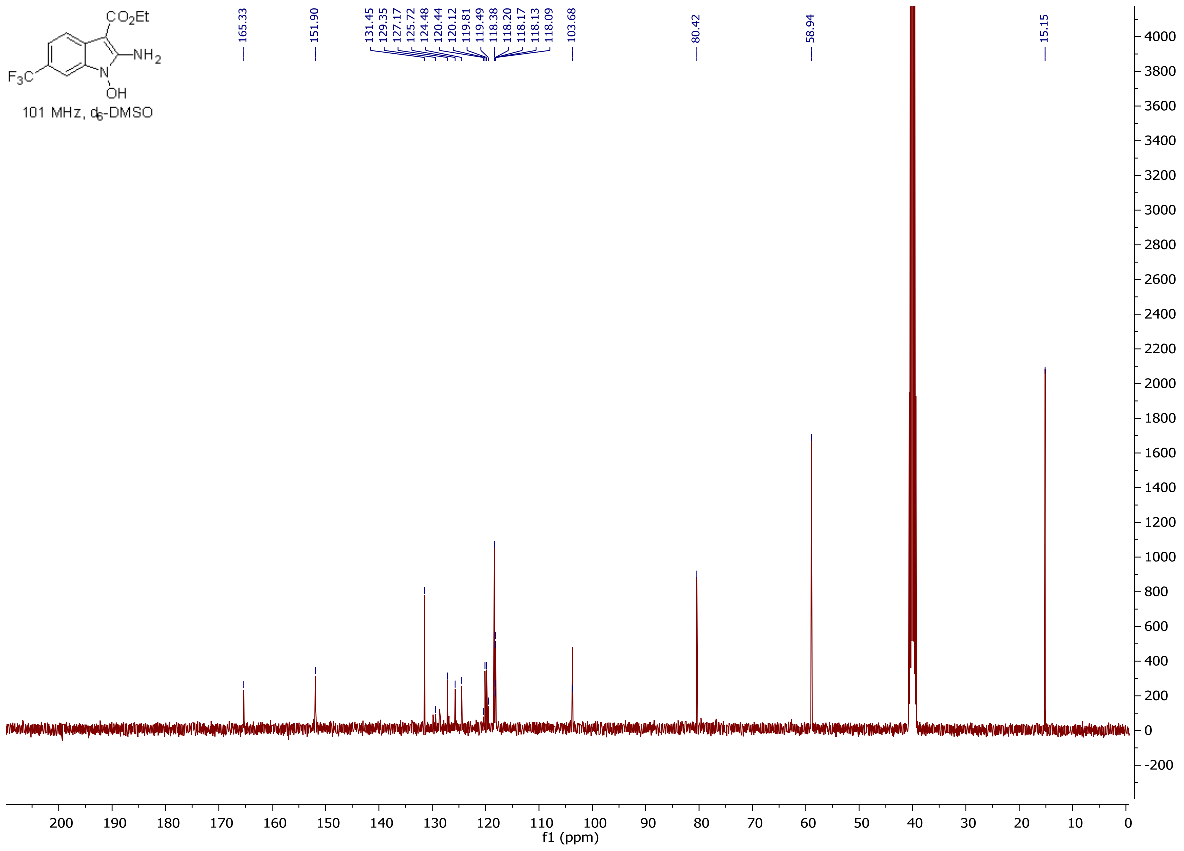
CO2Et
NH2
F3C
N
OH
101 MHz, d6-DMSO
165.33
151.90
131.45
129.35
127.17
125.72
124.48
120.44
120.12
119.81
119.49
118.38
118.20
118.17
118.13
118.09
103.68
80.42
58.94
15.15
f1 (ppm)

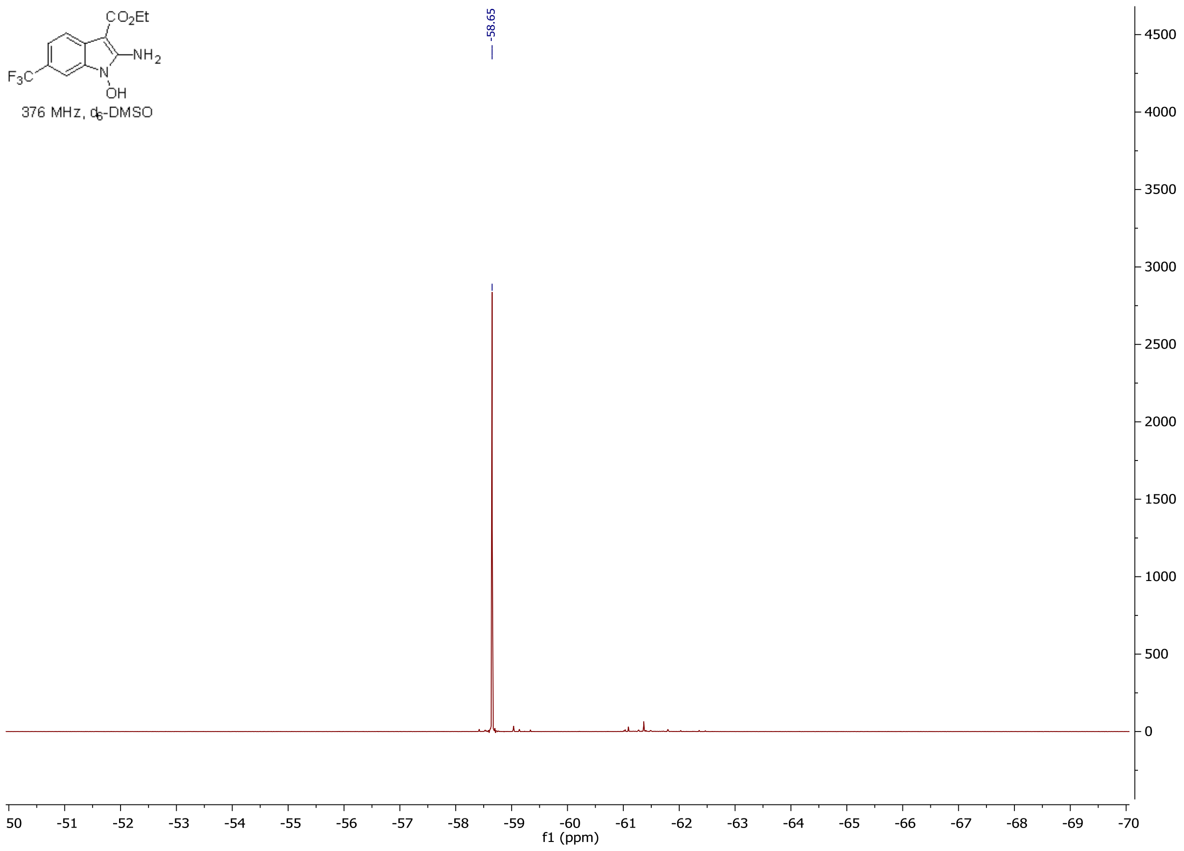
CO2Et
NH2
F3C
N
OH
376 MHz, d6-DMSO
-58.65
f1 (ppm)

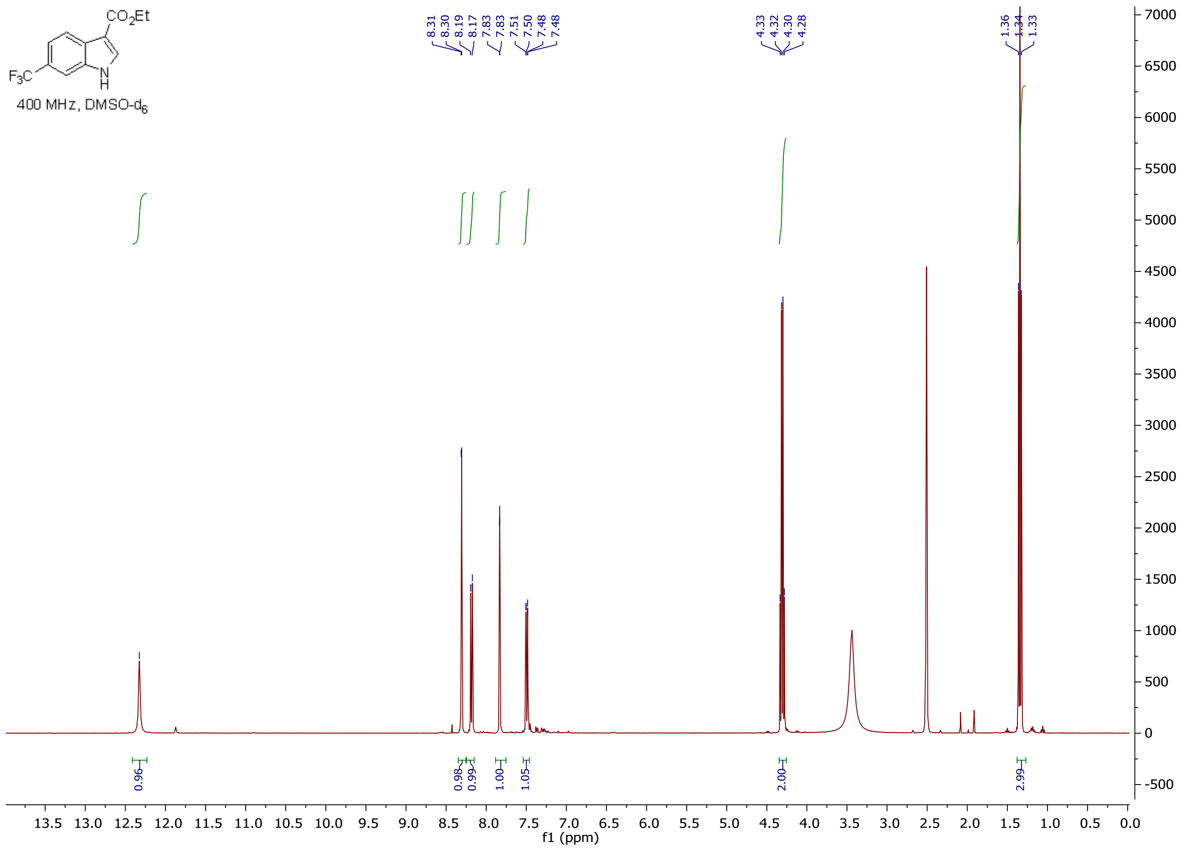
CO2Et
F3C
N
H
400 MHz, DMSO-d6
8.31
8.30
8.19
8.17
7.83
7.83
7.51
7.50
7.48
7.48
4.33
4.32
4.30
4.28
1.36
1.34
1.33
0.96
0.98
0.99
1.00
1.05
2.00
2.99
f1 (ppm)

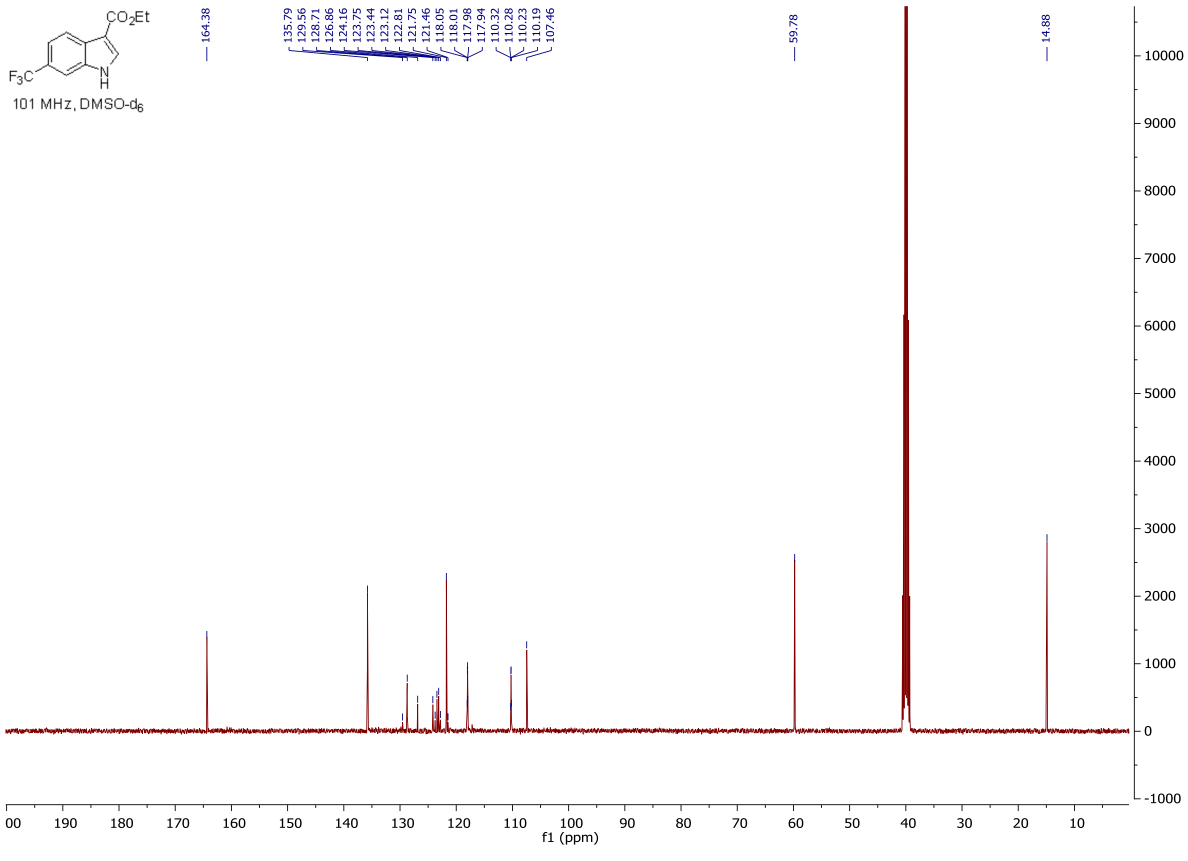
CO2Et
F3C
N
H
101 MHz, DMSO-d6
164.38
135.79
129.56
128.71
126.86
124.16
123.75
123.44
123.12
122.81
121.75
121.46
118.05
118.01
117.98
117.94
110.32
110.28
110.23
110.19
107.46
59.78
14.88
f1 (ppm)

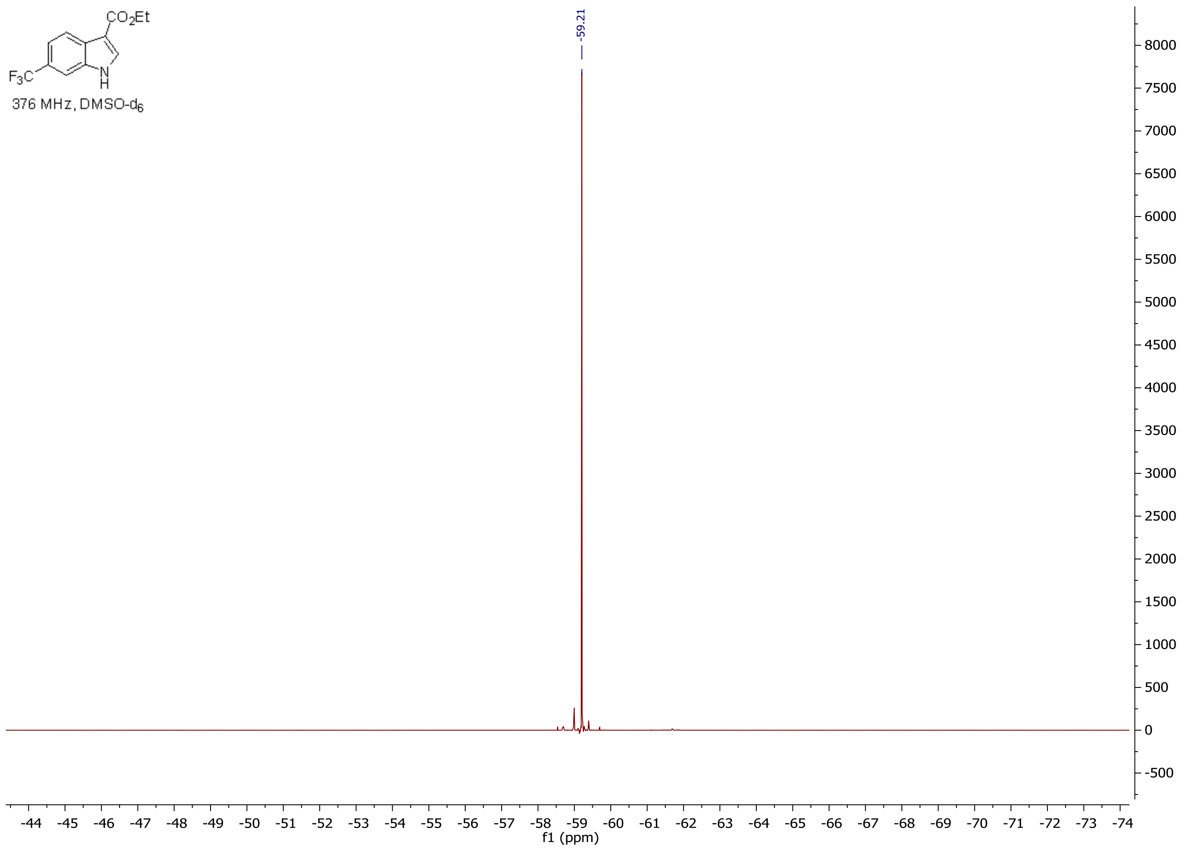
CO2Et
F3C
N
H
376 MHz, DMSO-d6
-59.21
f1 (ppm)

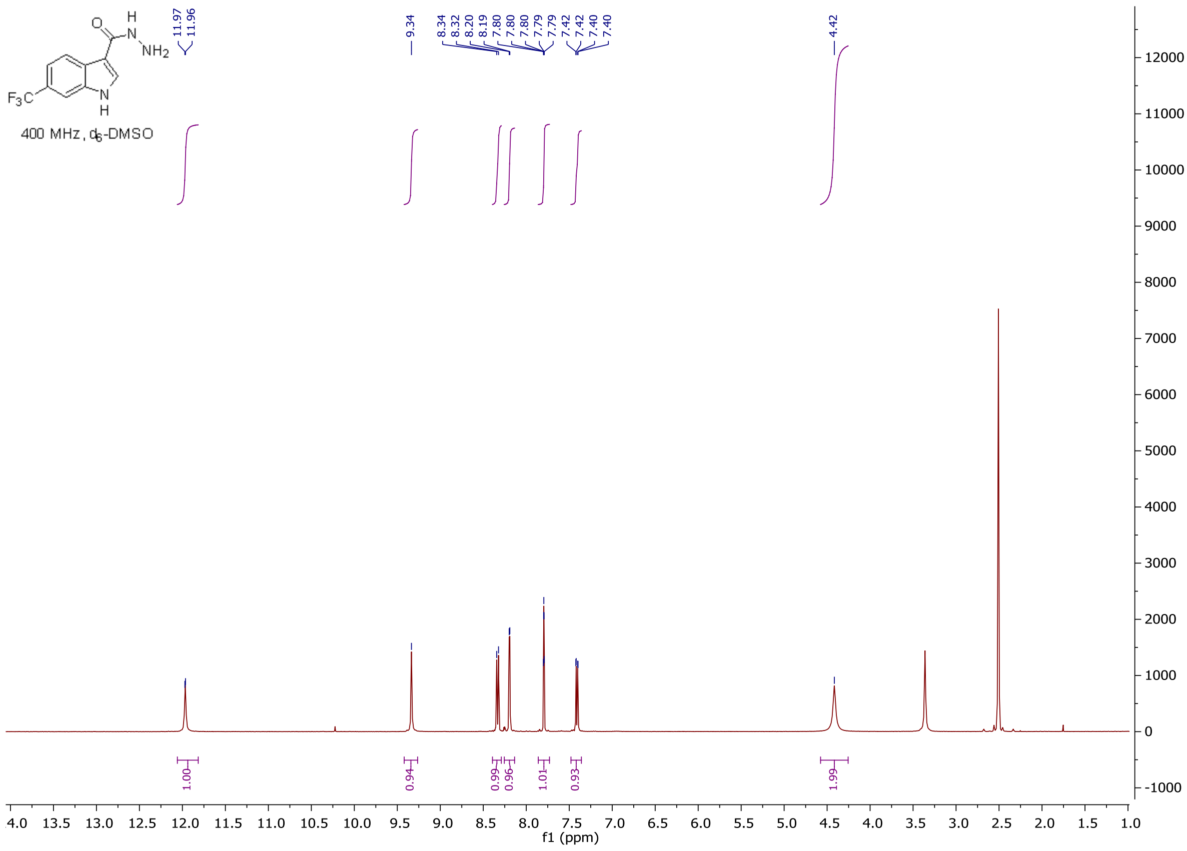
O
H
N
NH2
F3C
N
H
400 MHz, d6-DMSO
11.97
11.96
9.34
8.34
8.32
8.20
8.19
7.80
7.80
7.80
7.79
7.42
7.42
7.40
7.40
4.42
1.00
0.94
0.99
0.96
1.01
0.93
1.99
f1 (ppm)

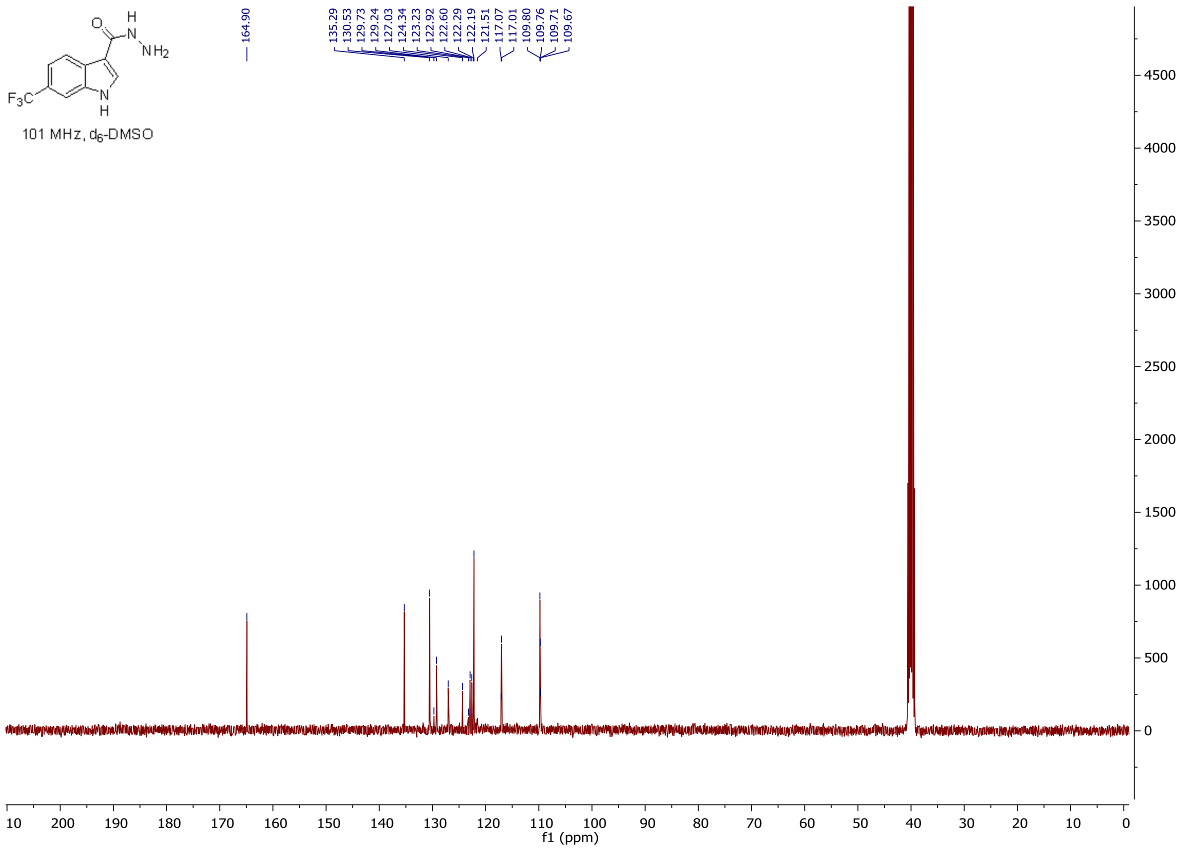
O
H
N
NH2
F3C
N
H
101 MHz, d6-DMSO
164.90
135.29
130.53
129.73
129.24
127.03
124.34
123.23
122.92
122.60
122.29
122.19
121.51
117.07
117.01
109.80
109.76
109.71
109.67
f1 (ppm)

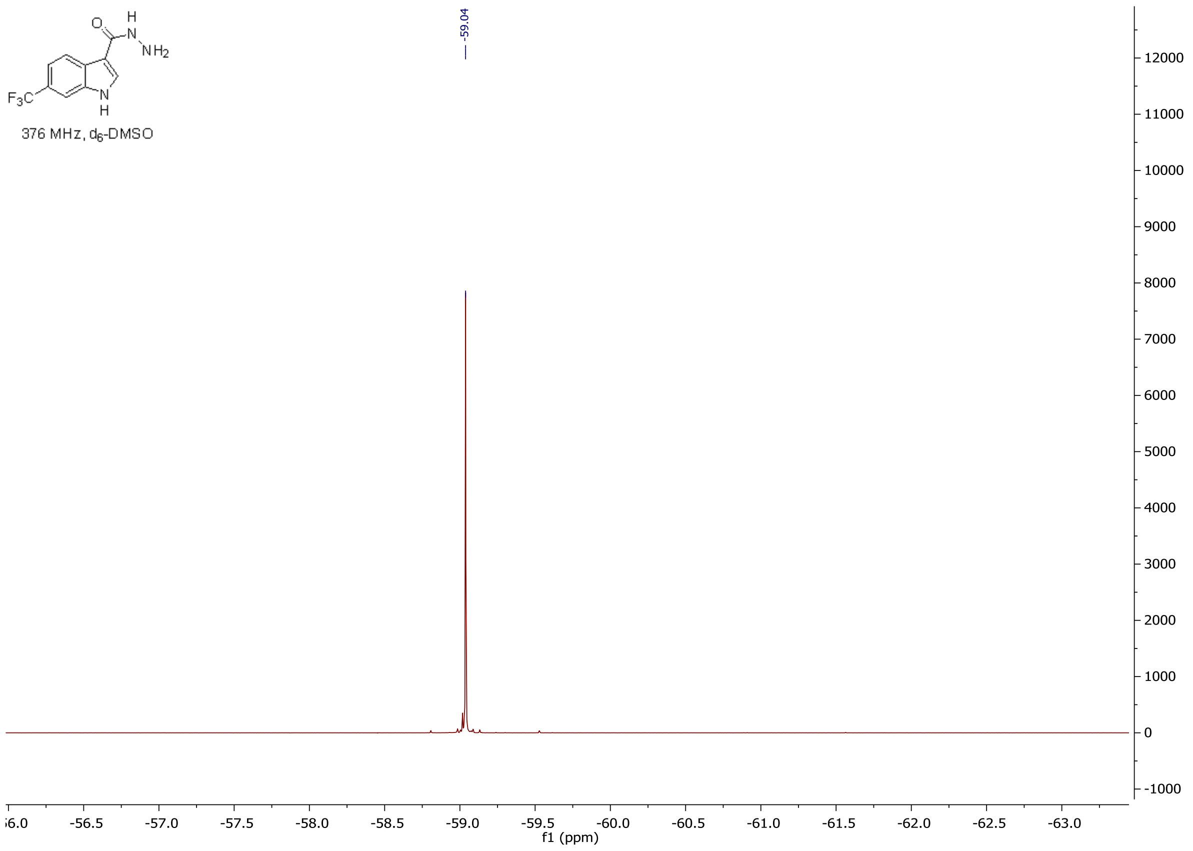
O
H
N
NH2
F3C
N
H
376 MHz, d6-DMSO
-59.04
f1 (ppm)

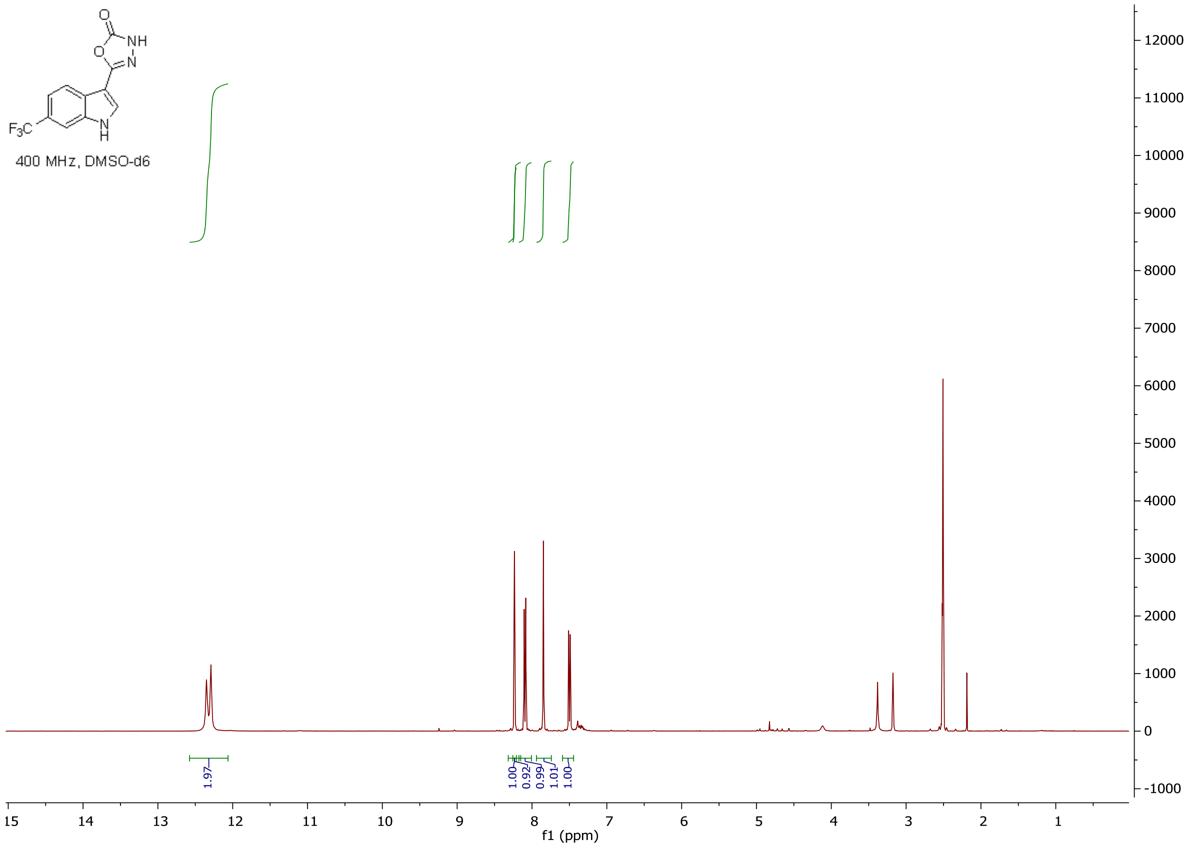

O
NH
O
N
F3C
N
H
400 MHz, DMSO-d6
1.97
1.00
0.92
0.99
1.01
1.00
f1 (ppm)

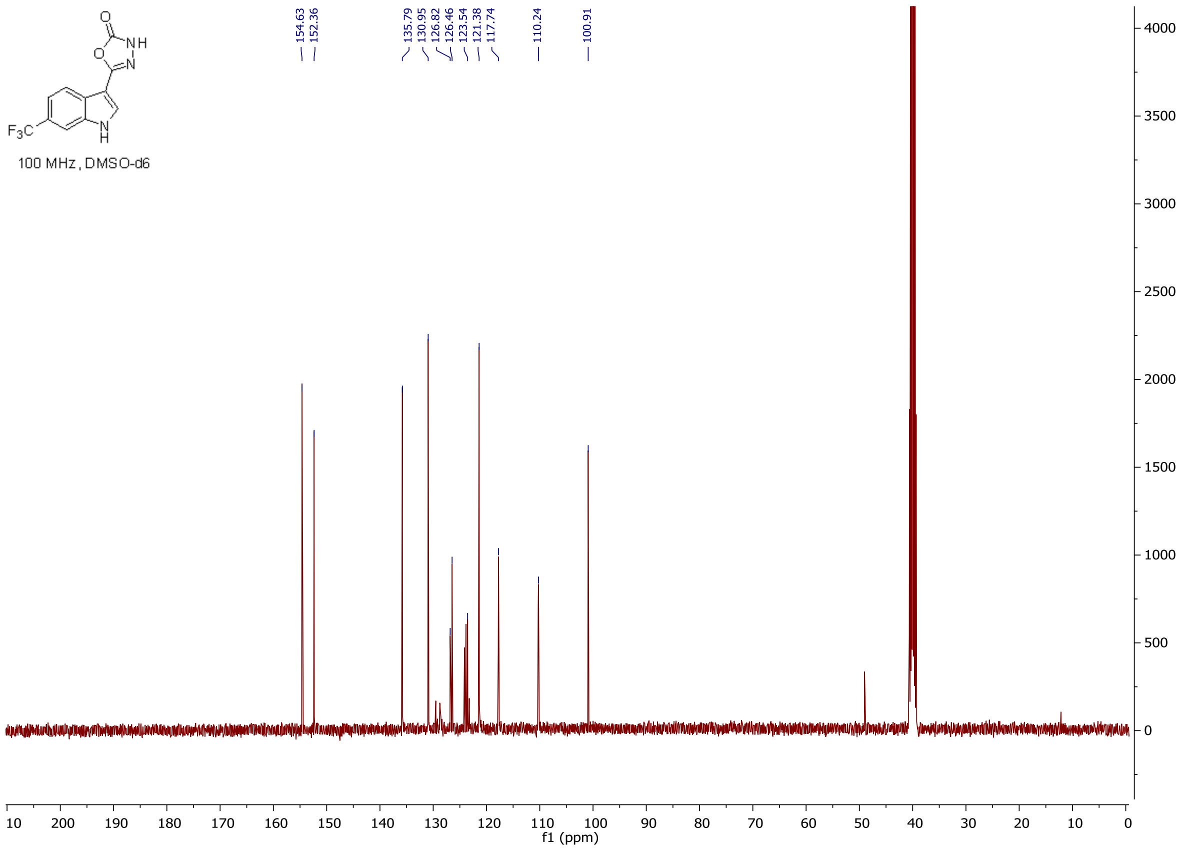

154.63
152.36
135.79
130.95
126.82
126.46
123.54
121.38
117.74
110.24
100.91
O
NH
O
N
F3C
N
H
100 MHz, DMSO-d6
f1 (ppm)
